# Efficacy and Safety of Auricular Acupuncture for Cognitive Impairment and Dementia: A Systematic Review

**DOI:** 10.1155/2018/3426078

**Published:** 2018-05-31

**Authors:** Chan-Young Kwon, Boram Lee, Hyo-Weon Suh, Sun-Yong Chung, Jong Woo Kim

**Affiliations:** ^1^Department of Clinical Korean Medicine, Graduate School, Kyung Hee University, Kyung Hee Dae-ro 26, Dongdaemun-gu, Seoul 02447, Republic of Korea; ^2^Department of Neuropsychiatry, Kyung Hee University Korean Medicine Hospital at Gangdong, Dongnam-ro 892, Gangdong-gu, Seoul 05278, Republic of Korea

## Abstract

**Objectives:**

To analyze the efficacy and safety of auricular acupuncture (AA) in patients with cognitive impairment and dementia.

**Methods:**

Twelve electronic databases were searched for randomized controlled trials evaluating effects of AA in patients with cognitive impairment and/or dementia, from their inception to August 2017. The primary outcome was cognitive function, and secondary outcomes were self-care ability, quality of life, clinical efficacy rate, and incidences of adverse events.

**Results:**

Nine studies were included, and five involving 677 participants were analyzed quantitatively. Compared with Western medications (WM), AA had mixed effects on cognitive functions (Mini-Mental State Examination [MMSE], mean difference [MD] 0.73, 95% confidence interval [CI] −0.02 to 1.48; Hierarchic Dementia Scale [HDS], MD 2.21, 95% CI 1.09 to 3.33); there was no significant improvement in the activities of daily living (ADL) score (MD 0.20, 95% CI −3.51 to 3.91) in patients with vascular dementia (VD). Compared to WM, AA combined with WM showed better clinical efficacy rate (risk ratio [RR] 1.42, 95% CI 1.06 to 1.91) in patients with VD; there was no significant improvement in cognitive functions (MMSE, MD 0.97, 95% CI −0.44 to 2.38; Montreal Cognitive Assessment [MoCA], MD 0.22, 95% CI −1.83 to 2.27) in patients with mild cognitive impairment (MCI). Compared to herbal medicine (HM), AA plus HM showed significant improvements in cognitive function (MMSE, MD 1.31, 95% CI 0.13 to 2.49) in patients with MCI and patients with vascular cognitive impairment, no dementia (VCIND) and in ADL score (MD −6.70, 95% CI −8.78 to −4.62) in patients with MCI. No adverse event associated with AA was reported.

**Conclusion:**

The evidence reveals mixed efficacy of AA in patients with cognitive impairment and/or dementia. However, the results were inconclusive because of the small number and poor methodological quality of the included studies.

## 1. Introduction

Dementia is a common neurodegenerative disease of the central nervous system globally [1, 2]. As the worldwide population ages, it is recognized that the disease burden worldwide will increase. According to the 2015 World Alzheimer Report, the incidence of dementia will reach 131.5 million by 2050 and the estimated burden of dementia in 2018 will reach a trillion dollars [2]. Alzheimer's disease (AD) and vascular dementia (VD) are representative types of dementia. Although the development of new drugs for these types of diseases is ongoing, there is no established treatment, making normal recovery very difficult once the dementia develops [3]. Thus, early detection of cognitive impairment, assessment of the risk of progression to dementia, and prevention of the progression are emphasized in the management of dementia to ease the socioeconomic burden for patients, their caregivers, and their medical teams [4, 5].

Mild cognitive impairment (MCI) refers to a cognitive state that is less than the expected level of cognitive function according to the individual's age or education level; it is not severe enough to interfere with activities of daily living (ADL) [6]. MCI can be described as the “predementia stage” and is the primary target for early detection and management of dementia. Medications such as rivastigmine and donepezil have been tried for the treatment of MCI, but there are limitations in terms of their efficacy and safety [7, 8]. Nonpharmaceutical treatments, such as transcranial magnetic stimulation, have been attempted as well [9], but there is currently no established treatment for MCI.

Auricular acupuncture (AA) is a safe nonpharmaceutical treatment that involves the application of a stainless steel needle or a medicinal herb on acupoints in the outer ear. This treatment originated in traditional Chinese medicine [10]; however, since the 1950s when Dr. Paul Nogier presented the outer ear as “an inverted fetus map” [11], it has become widely known in the West. It is now one of the most popular complementary and alternative medicine (CAM) therapies available in both the East and the West. AA is very tolerable and inexpensive, and its usefulness in many disorders or conditions, such as pain [12], constipation [13], addiction [14], and insomnia [15], has been examined. Furthermore, recent systematic reviews of the limited evidence suggest that acupuncture may improve cognitive function and ADL in patients with cognitive impairment and dementia [16, 17].

Although AA is a safe and cost-effective nonpharmaceutical treatment as described and can be used for the management and prevention of cognitive impairment and/or dementia, there has not been a systematic review of its efficacy and safety. Therefore, the aim of this review was to evaluate the efficacy and safety of AA in patients with cognitive impairment and dementia through a comprehensive review and meta-analysis.

## 2. Materials and Methods

This systematic review and meta-analysis was reported in accordance with the Preferred Reporting Items for Systematic Reviews and Meta-Analyses (PRISMA) guidelines [18]. The protocol of this review was registered in PROSPERO (CRD42017081646).

### 2.1. Search Strategy

We extensively searched the following English, Chinese, and Korean databases from inception to August 16, 2017: six English databases (MEDLINE via PubMed, Cochrane Central Register of Controlled Trials [CENTRAL], EMBASE via Elsevier, Allied and Complementary Medicine Database [AMED] via EBSCO, Cumulative Index to Nursing and Allied Health Literature [CINAHL] via EBSCO, and PsycARTICLES); two Chinese databases (China National Knowledge Infrastructure [CNKI] and Wanfang Data); and four Korean databases (KoreaMed, Koreanstudies Information Service System [KISS], Oriental Medicine Advanced Searching Integrated System [OASIS], and Korean Traditional Knowledge Portal [KTKP]). We also reviewed the reference lists of relevant articles to identify additional trials. There was no restriction on language or publication date. The search terms for each database are available in Supplementary Material 1.

### 2.2. Inclusion and Exclusion Criteria

#### 2.2.1. Types of Studies

We included only randomized controlled trials (RCTs) that assessed the beneficial effects of AA in patients with cognitive disorders. RCTs using quasi-random methods, such as alternate allocation or allocation by birth date, were also included.

#### 2.2.2. Types of Participants

Studies involving participants with all types of cognitive disorders, including AD, VD, MCI, and vascular cognitive impairment, no dementia (VCIND), diagnosed using standardized criteria such as the diagnostic and statistical manual of mental disorders (DSM), international classification of diseases (ICD), or Chinese classification of mental disorders (CCMD), were included. In addition, studies involving participants screened using validated cognitive assessment tools, such as the Mini-Mental State Examination (MMSE), were included. There were no limitations of age, sex, or race. Studies were excluded if participants had other serious illnesses such as cancer, liver disease, or kidney disease.

#### 2.2.3. Types of Interventions

We included AA as an experimental intervention. In this review, AA indicates not only the application of a needle penetrating into acupoints, but also the acupressure, which is a technique to press acupoints noninvasively with a finger or noninvasive tool such as a medicinal herb. Studies involving AA combined with other therapies as experimental interventions were also included if the other therapies were used equally in both the experimental and the control groups. Studies comparing different types of AA were excluded. There were no other restrictions in the control intervention.

#### 2.2.4. Types of Outcome Measures

The primary outcome was change in cognitive function as measured by validated assessment tools, including the MMSE, Montreal Cognitive Assessment (MoCA), Alzheimer's Disease Assessment Scale-Cognition (ADAS-Cog), Global Deterioration Scale (GDS), Clinical Dementia Rating (CDR), and Hierarchic Dementia Scale (HDS). The secondary outcomes included changes in self-care ability (measured by ADL) and quality of life (QoL). The clinical efficacy rate and the incidences of adverse events were also defined as secondary outcomes.

### 2.3. Study Selection

Two researchers (C.Y. Kwon and B. Lee) independently screened the titles and abstracts of the searched articles and performed eligibility assessment. Then, the two researchers reviewed the full texts of the selected articles. We excluded articles from the meta-analysis if they did not provide the statistical values required to perform the meta-analysis adequately. Any disagreement on study selection was resolved through discussion with other researchers.

### 2.4. Data Extraction

Two researchers (C.Y. Kwon and B. Lee) independently extracted data using predefined data collection forms. The extracted items included information about the participants, interventions, outcomes, results, and safety of the interventions, as well as information related to risk of bias, such as randomization and blinding methods. Any disagreement on data extraction was resolved through discussion with other researchers.

### 2.5. Risk of Bias Assessment

To assess the methodological quality of the included studies, two independent researchers (C.Y. Kwon and B. Lee) used the Cochrane's risk of bias tool, which is used to determine the methodological quality of RCTs by assessing the selection bias, performance bias, detection bias, attrition bias, reporting bias, and other potential biases. Because baseline imbalance in factors that are strongly related to outcome measures can cause other potential biases in the estimation of an intervention effect in RCTs, we assessed these factors (such as participant characteristics, including mean age, baseline MMSE score, and others) with particular emphasis on baseline imbalance between the experimental and the control groups [19]. Each type of bias received one of the following scores: “low risk,” “unclear risk,” or “high risk.” Any disagreement was resolved through discussion with other researchers.

### 2.6. Data Analysis

Descriptive analyses of the details of participants, interventions, outcomes, and results were performed for all included studies. For studies using the same type of experimental and control interventions and outcome measures, a meta-analysis was conducted using the Review Manager software, version 5.3 (Cochrane, London, United Kingdom). The results were pooled using a random-effect model if the included studies had significant heterogeneity, whereas a fixed-effect model was used when the heterogeneity was not significant. The fixed-effect model was also used when the number of studies included in the meta-analysis was very small, where the estimate of the between-study variance had poor precision [20]. The pooled data was described as a mean difference (MD) for continuous outcomes and a risk ratio (RR) for binary outcomes with 95% confidence intervals (CIs). The Chi-squared test and* I*-squared statistic were used to assess heterogeneity between studies, with an* I*-squared statistic >50% and >75% indicative of substantial and considerable heterogeneity, respectively [21].

### 2.7. Subgroup Analysis

Subgroup analysis was performed according to the participants' diseases or types of Western medication (WM) by its ingredients.

### 2.8. Publication Bias

If more than 10 studies were included in the meta-analysis, a funnel plot was used to evaluate publication bias.

## 3. Results

### 3.1. Study Selection

**Figure 1:**
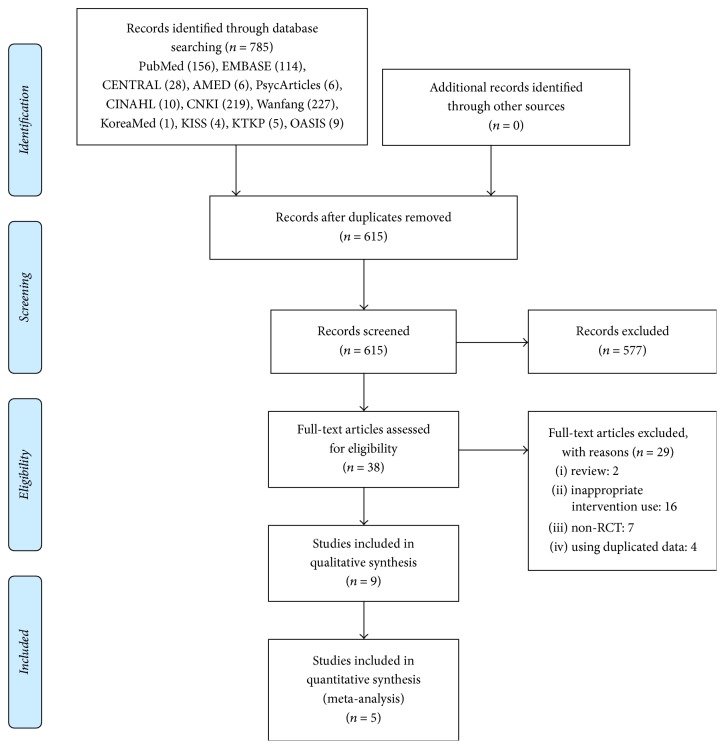
PRISMA flow chart of the study selection process.

The database search identified 785 potentially relevant studies, and no additional records were identified through other sources. After removing duplicates, 615 records remained. A total of 577 studies were excluded by screening the titles and abstracts, and the full texts of the remaining 38 articles were obtained and reviewed. Ultimately, nine studies [22–30] were included in the systematic review, and among them, five [22–24, 28, 30] were included in the meta-analysis (Figure 1).

### 3.2. Characteristics of the Included Studies

A summary of included studies is presented in Table 1. All studies were parallel study designs: five [23, 24, 26, 27, 30] were 3-arm parallel studies and four [22, 25, 28, 29] were 2-arm parallel studies. Two studies [24, 26] were theses for master's degrees. Two studies [26, 27] were approved by an institutional review board (IRB) before the studies were started, and six studies [22–27] reported that an informed consent form from the participants was received. Eight studies [22–26, 28–30] were conducted in China and one [27] was conducted in Spain. Although 997 subjects participated in the included studies, the number of participants analyzed in one study [29] was not provided; therefore it was not established how many were included in the analysis of this review. Four studies [22–25] were conducted in patients with VD and one [30] was conducted on patients with VCIND. Participants with MCI [26, 28] and unspecified dementia [27, 29] were each included in two studies.

In all studies, AA was used as the experimental intervention. In two studies [23, 24], the experimental group consisted of two groups: both AA and AA plus moxibustion were used as experimental interventions. Auricular* shen men *[22, 25–30], kidney [22, 24–26, 28–30], and heart [24–30] points were used most frequently (seven studies). Seven studies [22–28] used* Vaccaria *seeds as AA material, and the remaining two studies [29, 30] did not mention the specific AA material used. The number of self-acupressure sessions recommended ranged from two to five per day. The most frequent treatment periods were 12 weeks [22–24] and three months [25, 27, 28] (each in three studies), and follow-up assessment was performed in only two studies, one at 12 weeks [24] and the other one at two months [27]. Details of AA methods are presented in Table 2. For control interventions, four studies [22–25] used WM and two studies [28, 29] used herbal medicine (HM). In three studies, the control consisted of two groups; the following combinations were used: WM and no intervention [26]; routine care plus relaxing massage and routine care alone [27]; and HM and WM [30].

Cognitive function was evaluated in eight studies [22–26, 28–30] using one or more of the following outcome measures: MMSE (seven studies) [22–24, 26, 28–30], MoCA (two studies) [26, 30], HDS (two studies) [23, 24], and clinical efficacy rate (one study) [25]. Self-care ability was assessed in five studies [22, 24, 25, 28, 29]: ADL in four studies [22, 24, 28, 29]; and clinical efficacy rate in one study [25]. There were no studies evaluating QoL or cognitive function using ADAS-Cog, GDS, or CDR. Five studies [22–24, 26, 28] reported the incidence of adverse events associated with AA.

### 3.3. Risk of Bias Assessment

**Figure 2:**
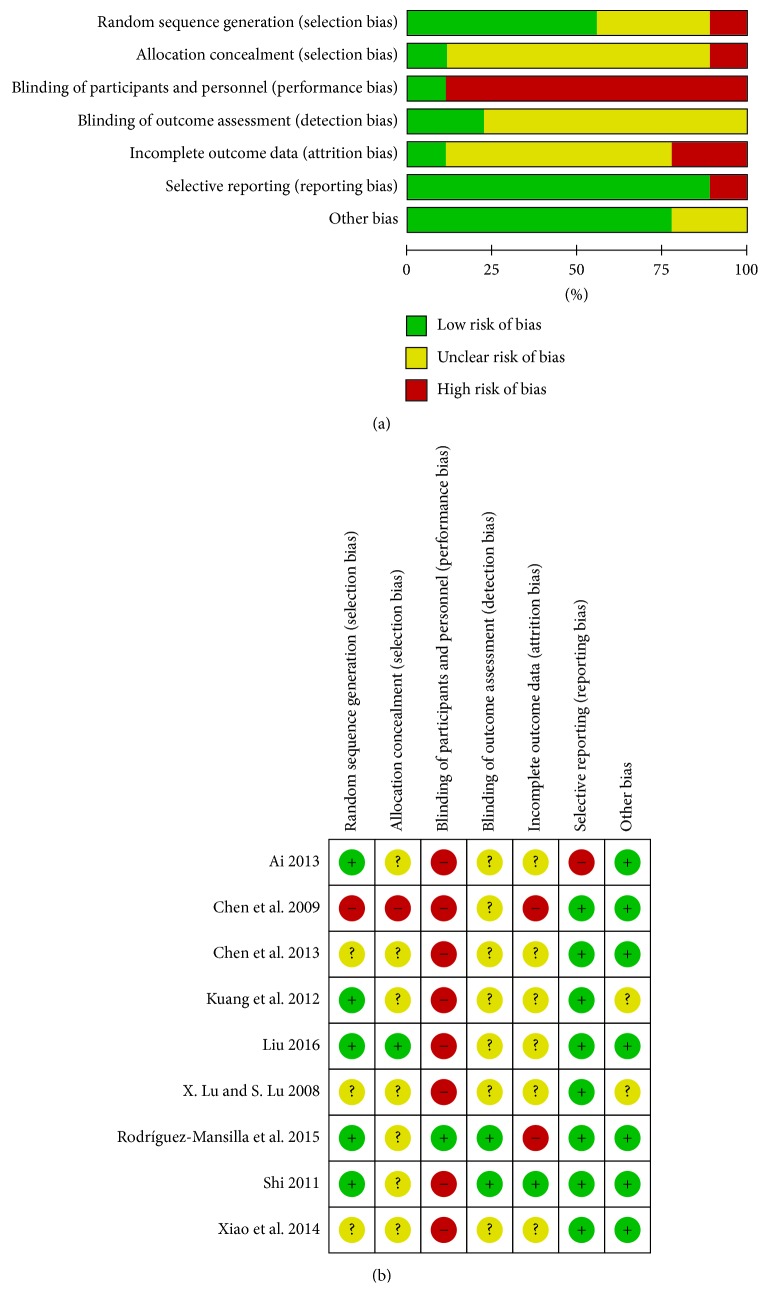
Risk of bias: (a) risk of bias graph and (b) risk of bias summary; “+” is low risk, “?” is unclear risk, and “−” is high risk.

In terms of random sequence generation, five studies [23–27] using random sequences, such as random number table, were classified as “low risk”; one study [22] with sequence generation using the order of treatment was rated as “high risk”; and three studies [28–30] with no relevant information were rated as “unclear.” One study [26] using sealed envelopes for allocation concealment was rated as “low risk.” Except for one study [27] that performed intervention blinding of participants and personnel, all the studies were rated as “high risk” with respect to performance bias because of the nature of the AA intervention. Blinding of an outcome assessor was conducted in two studies [24, 27], which were classified as “low risk” with respect to detection bias. Two studies [22, 27] that conducted per-protocol analysis and one study [25] that reported only clinical efficacy rate without presenting the raw data were classified as “high risk” with respect to both attrition and reporting bias. Seven studies [22, 24–28, 30] that reported no statistically significant demographic differences between experimental and control group at baseline were classified as “low risk” with respect to other biases. Results of the risk of bias assessment of the nine RCTs [22–30] are presented in Figure 2.

### 3.4. Auricular Acupuncture versus Western Medications

Three studies [22–24] compared AA with WM; two of them [23, 24] were 3-arm parallel studies that used both AA plus moxibustion and AA alone as experimental interventions. In all three studies, the participants were diagnosed with VD, and the treatment period was 12 weeks.

#### 3.4.1. Cognitive Function

**Figure 3:**
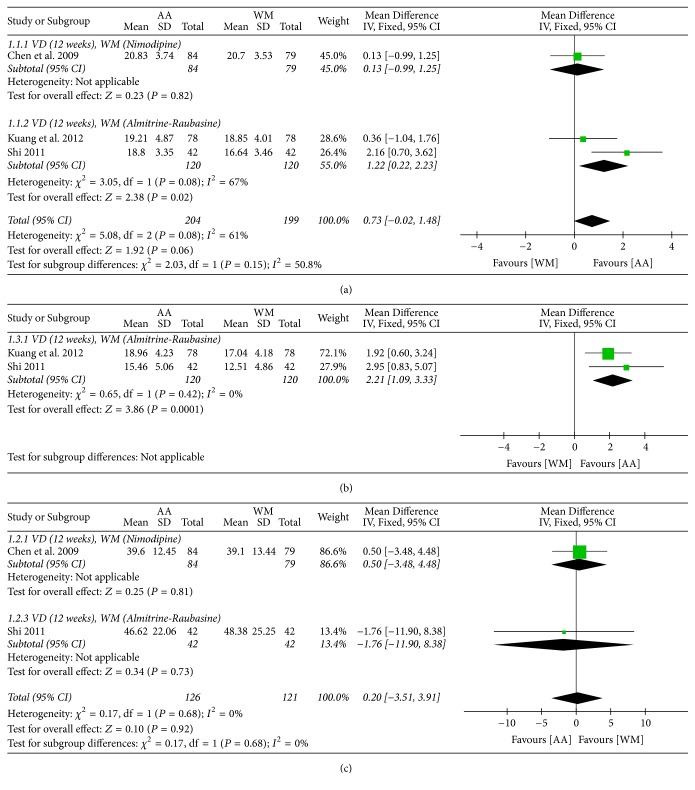
Forest plots for comparison of auricular acupuncture (AA) versus Western medications (WM). (a) Mini-Mental State Examination (MMSE), (b) Hierarchic Dementia Scale (HDS), and (c) activities of daily living (ADL).

All studies evaluated the MMSE score after an intervention of 12 weeks, and the pooled results showed that there was no significant difference between AA and WM (MD 0.73, 95% CI −0.02 to 1.48, and *I*^2^ = 61%). However, subgroup analysis, according to the types of WM used, showed significant favorable results for AA when the control group was treated with almitrine and raubasine (MD 1.22, 95% CI 0.22 to 2.23, and *I*^2^ = 67%) [23, 24], but not for nimodipine (MD 0.13, 95% CI −0.99 to 1.25) [22]. The meta-analysis showed that the improvement in HDS scores after the intervention was significantly higher in the AA group than in the WM group, and there was no heterogeneity (MD 2.21, 95% CI 1.09 to 3.33, and *I*^2^ = 0%) [23, 24] (Figures 3(a) and 3(b)).

#### 3.4.2. Self-Care Ability

Two studies [22, 24] evaluated self-care ability using the ADL score after an intervention of 12 weeks. The pooled results showed no difference between AA and WM (MD 0.20, 95% CI −3.51 to 3.91, and *I*^2^ = 0%) (Figure 3(c)).

### 3.5. Auricular Acupuncture Plus Western Medication versus Western Medication Alone

Three studies [25–27] compared AA plus WM to WM alone; two of these [26, 27] were 3-arm parallel studies that used the wait-list and routine care plus relaxing massage as additional comparisons. The diagnoses included in this comparison were VD [25], MCI [26], and dementia [27], and the treatment periods varied from 3 to 12 months. Due to the heterogeneity of the reported outcomes between the studies, only a qualitative analysis was performed.

#### 3.5.1. Cognitive Function

Two studies [25, 26] reported outcomes related to cognitive function. One study [26] evaluated cognitive function using MMSE and MoCA in participants with MCI. MMSE and MoCA scores were not significantly different between AA plus WM and WM alone after an intervention of 12 months (MD 0.97, 95% CI −0.44 to 2.38; MD 0.22, 95% CI −1.83 to 2.27, respectively). The other study [25] assessed the clinical efficacy rate in participants with VD using symptom improvement, cognitive function, and self-care ability. The results showed that when AA was added to WM, the clinical efficacy rate was significantly higher compared to that of WM alone after an intervention of three months (RR 1.42, 95% CI 1.06 to 1.91).

### 3.6. Auricular Acupuncture Plus Herbal Medicine versus Herbal Medicine Alone

There were three studies [28–30] comparing AA plus HM to HM alone; one [30] was a 3-parallel study that evaluated both HM and WM as control groups. The diagnoses included in this comparison were MCI [28], dementia [29], and VCIND [30], and the treatment period varied from 45 days to six months.

#### 3.6.1. Cognitive Function

**Figure 4:**
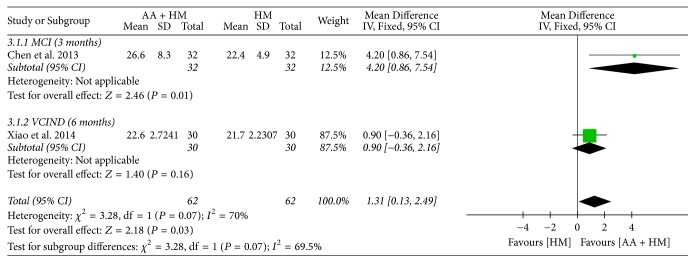
Forest plots for comparison of auricular acupuncture (AA) plus herbal medicine (HM) versus HM alone. Outcome: Mini-Mental State Examination (MMSE).

All studies assessed cognitive function using MMSE; one study [29] that involved participants with dementia did not present the standard deviation value. In a meta-analysis of the remaining two studies [28, 30], the AA plus HM group showed a significantly higher MMSE score than did the HM alone group; there was significant heterogeneity, which might have been caused by the participants' disease (MD 1.31, 95% CI 0.13 to 2.49, and *I*^2^ = 70%) (Figure 4). One study [30], which involved participants with VCIND, evaluated MoCA scores and found that the AA plus HM group showed significant improvement in MoCA scores compared with the scores in the HM group after an intervention of six months (MD 1.77, 95% CI 0.60 to 2.93). In the study [29] that was not included in the meta-analysis, the mean MMSE score of participants with dementia was improved in the AA plus HM group compared to the one in the HM alone group after an intervention of 45 days; however, no statistical test comparing the two groups was performed (MMSE score: 26 versus 22).

#### 3.6.2. Self-Care Ability

Two studies [28, 29] evaluated self-care ability using ADL scores after the intervention. The study [28] that involved participants with MCI showed a significant reduction in ADL scores in the AA plus HM group compared to the HM alone group after an intervention of three months (MD −6.70, 95% CI −8.78 to −4.62). In the other study [29], the mean ADL score of participants with dementia was lower in the AA plus HM group compared to the one in the HM alone group after an intervention of 45 days; however, no statistical test comparing the two groups was performed (ADL score: 30 versus 40).

### 3.7. Other Effects of Auricular Acupuncture

One study [27] evaluated pain, depression, and anxiety in participants with dementia receiving AA plus routine care or relaxing massage plus routine care. These symptoms were evaluated using the Doloplus-2 scale, Cornell scale for depression in dementia (CSDD), and the Campbell scale. The results showed that, with respect to pain and depression, the improvements from baseline scores were significantly higher in the experimental group than in the control group at one, two, and three months of treatment and at one month of follow-up assessment (*P* < 0.01 or *P* < 0.05), but there were no differences at two months of follow-up assessment (*P* > 0.05). With respect to anxiety, the improvement was significantly higher in the experimental group at one month of follow-up assessment (*P* < 0.05), but there were no differences at one, two, and three months of treatment and at two months of follow-up assessment (*P* > 0.05 for all).

The other study [30], which involved participants with VCIND, evaluated the serum level of 25-hydroxyvitamin D after the interventions, and the results showed that the AA plus HM and HM alone groups had significantly higher levels than did the WM alone group after an intervention of six months (*P* < 0.01 for both).

### 3.8. Safety

Five studies [22–24, 26, 28] described adverse events that occurred during treatment. Four studies [23, 24, 26, 28] reported that there were no adverse events during the interventions, and one study [22] reported that one case of mild dizziness and one case of diarrhea occurred in the control group using WM. No adverse events associated with AA were reported among the studies included in this review.

### 3.9. Publication Bias

Analysis of funnel plots is a practical test to detect potential publication bias in systematic reviews. However, the number of studies included in this review was only nine, so publication bias could not be assessed.

## 4. Discussion

This systematic review was conducted to evaluate the efficacy and safety of AA in patients with cognitive impairment and dementia. In total, nine RCTs were included after comprehensive searches, and five of these, involving 677 participants, were analyzed quantitatively. There were three kinds of comparisons: AA versus WM; AA plus WM versus WM; and AA plus HM versus HM.

Three studies [22–24] compared AA versus WM treatments for 12 weeks in patients with VD. Compared to WM, AA showed a significant improvement in cognitive function of participants as assessed by HDS, whereas AA had mixed effects on MMSE, depending on the subgroup analysis according to the type of WM used. Notably, AA was significantly superior to some WMs, such as almitrine and raubasine, but not nimodipine. However, it is unclear whether this difference is because of the difference in the content of WM. The study [22] using nimodipine as a control reported that the baseline MMSE score was 18.00 ± 3.88 in the AA group and 17.80 ± 3.82 in the WM group, whereas the two studies [23, 24] using almitrine and raubasine as a control reported that baseline MMSE was 16.06 ± 2.86, 16.59 ± 3.68, respectively, in the AA group and 15.32 ± 2.48, 16.77 ± 3.21, respectively, in the WM group. Since the minimum clinically important difference for MMSE is 1.4 points [31], these differences in baseline MMSE scores indicated that the degree of cognitive impairment of participants was different between the study using nimodipine and the studies using almitrine and raubasine. Therefore, the significant results of the subgroup analysis may be influenced not only by the type of WM, but also by the baseline degree of cognitive impairment of the participants. There was no significant difference in the improved ADL scores between AA and WM. However, this result may be caused by not only the difference in the type of WM used, but also the difference in the baseline ADL scores; this is because a study [22] using nimodipine as a control WM reported that the mean baseline ADL score was 55.42 ± 22.16 in the AA group and 58.51 ± 25.35 in the WM group. The other study [24] using almitrine and raubasine as control WMs reported that the mean baseline ADL score was 44.90 ± 14.84 in the AA group and 45.70 ± 14.86 in the WM group. Compared to WM, AA plus WM in the treatment of VD for three months showed a significantly higher clinical efficacy rate determined based on dementia symptoms, cognitive function, and self-care ability.

AA plus WM for 12 months did not show a significant difference in improving cognitive function as assessed by MMSE and MoCA compared to WM alone in patients with MCI [26]. However, AA plus HM for three months [28] or six months [30] showed a significant improvement in cognitive function as assessed by MMSE or MoCA compared to HM alone in participants with MCI or VCIND. Moreover, AA plus HM for three months [28] showed a significant effect in reducing ADL scores compared to HM alone in participants with MCI.

Although the clinical mechanisms of AA in improving cognitive impairment and/or major symptoms of dementia have not yet been established, several hypotheses can be suggested. First, AA can improve cognitive function through neuroprotective effects. In AD model rats, AA promoted the expression of choline acetyltransferase (ChAT) in the hippocampus and decreased the expression of glial fibrillary acidic protein (GFAP) [32]. This suggests that AA can participate in acetylcholine synthesis and regulate abnormal astrocytic hyperactivity. Furthermore, it has been suggested that electrical stimulation of the ears of rats with cerebral ischemia-reperfusion injury has a neuroprotective effect by promoting the secretion of acetylcholine [33]. In VD model rats, AA could upregulate bcl-2 expression in brain tissues, suggesting that AA may have a neuroprotective effect by modulating apoptosis [34]. Moreover, short-term AA has been shown to improve antioxidant capacity in people at high risk for diabetes, suggesting that AA may reduce the risk for dementia, and it may have an indirect neuroprotective effect through the control of the body's antioxidant capacity [35]. Second, AA can directly affect the expression of beta-amyloid protein. In VD model rats, AA significantly improved learning and memory capacity, and inhibition of overproduction of beta-amyloid protein in brain was proposed as the mechanism [36]. Third, AA can prevent or improve cognitive impairment and/or dementia directly or indirectly through the “ear-vagus nerve reflex” [37]. Two pilot studies [38, 39] have shown that long-term auricular vagus nerve stimulation, a kind of electroacupuncture at the auricular branch of the vagus nerve, improved ADAS-Cog and MMSE scores of participants with AD. Based on these results, it has been suggested that AA may be effective in preventing and treating neurodegenerative diseases through a mechanism that activates the vagal nuclei in the brainstem [40]. AA can also indirectly prevent dementia by controlling the risk factors for dementia through its cardiovascular benefits [41, 42]. Finally, treatment procedures associated with AA may have served as a form of cognitive training. Six studies [22–25, 28, 30] included in this review requested participants to perform self-acupressure several times each day. This can be regarded as a task requiring participants to use memory recall. However, this is only a hypothesis, and further research is required to determine its validity.

From a medical point of view, treatments and/or management of dementia and its prevention (which includes the management of MCI) require a long-term care period rather than short-term intensive treatment. Therefore, compliance, economic costs, safety, and efficacy of the interventions are important. Though acupuncture is often associated with some adverse events, such as local pain, infection, dizziness, or syncope, serious adverse events are generally considered rare, indicating that acupuncture is one of the safest nonpharmaceutical treatments [43, 44]. Furthermore, the cost-effectiveness of acupuncture has been demonstrated [45]. In particular, AA has been used effectively to improve the mental health of people facing disasters in developing countries because of its cost-effectiveness [46]. To this end, the results of this first systematic review suggest the applicability of AA in the treatment of cognitive impairment and dementia. These results can be used not only for patients and their caregivers, but also for socioeconomic reasons to reduce disease burden.

Despite these strengths of AA, there are several limitations to this analysis. First, the methodological quality of the included RCTs, which was assessed using Cochrane's risk of bias tool, was generally poor. In terms of selection bias, performance bias, detection bias, and attrition bias, most studies had high or uncertain risk of bias. In particular, the blinding of participants, personnel, and assessors was not performed or described in most cases, suggesting that the results of the studies may be greatly affected by the placebo effect or overestimated by the assessors. Second, the results of the included RCTs could have a barrier to generalization as the studies were all conducted in China, except for the one conducted in Spain [27]. Third, only nine studies were included in this review, and the sample sizes were all small. In addition, only two studies [24, 27] followed the participants over a long period of time to monitor for the outcomes, such as the occurrence of AD or the decline of cognitive function over a significant period. Fourth, because of the small number of studies included and the heterogeneity of the treatment procedures, no optimal treatment protocol for the prevention and treatment of cognitive impairment and/or dementia could be derived. Finally, though AA has been considered a relatively safe treatment in past reviews, its safety in the treatment of cognitive impairment and/or dementia was not conclusive because only five studies [22–24, 26, 28] reported adverse events in this review.

In future studies, the recommendations outlined below should be considered. (1) RCTs with a large sample size and rigorous study design should be undertaken. (2) Long-term follow-up should be conducted to include the incidence of dementia and the rate cognitive decline. In particular, in the subjects with MCI, the rate of transition to dementia can be an important long-term outcome. (3) The economic efficiency of AA should be evaluated in the prevention and management of cognitive impairment and/or dementia. (4) Various AA methods should be considered. In particular, electrical stimulation of auricular acupoints, which has been well documented on an experimental basis, should be evaluated for efficacy and safety in different populations. (5) Effects other than on cognitive function, such as effects on the behavioral and psychological symptoms of dementia, which make it difficult to manage dementia, should be examined.

## 5. Conclusion

The results of this systematic review provide limited evidence for the efficacy of AA in improving cognitive function and self-care ability in patients with cognitive impairment and/or dementia. However, because the number of studies included was small and the methodological quality was generally poor, the results are not conclusive. Subsequent larger and more rigorous RCTs should be performed to confirm the efficacy and safety of AA.

## Figures and Tables

**Table 1:** Characteristics of included studies.

Study ID	Sample size (included →analyzed)	Mean age (range, years)/Sex (M : F)	Population	(A) Experimental intervention	(B) Control intervention	Outcomes	Results (*P* value)	Adverse events
*AA vs WM*								

Chen et al. 2009 [22]	180 (90 : 90) →163 (84 : 79)	(A) 70.41 ± 7.32 (58–82)/48 : 36 (B) 71.56 ± 6.27 (62–84)/40 : 39	Dementia (DSM-IV-R) VD (NINDS-AIREN) History of stroke More than 3 months continued dementia MMSE 12–24 HIS ≥ 7 CSDD < 8	AA	WM (Nimodipin 30 mg tid)	(1) MMSE (2) ADL	(1) N.S (2) N.S	(B) mild dizziness 1, diarrhea 1
Kuang et al. 2012 [23]	234 (78 : 78 : 78) →234 (78 : 78 : 78)	NR/NR	VD (DSM-IV-R)	(A1) AA (A2) AA + Moxa	WM (Almitrine-Raubasine tablets 1T bid)	(1) TCM symptoms (2) Clinical efficacy rate (TCM symptoms) (3) MMSE (4) HDS	(1) (A1) < (B)^+^, (A2) < (B)^+^ (2) (A1) > (B)^*∗*^, (A2) > (B)^*∗*^, (A2) > (A1)^*∗*^ (3) (A1) > (B)^*∗*^, (A2) > (B)^*∗*^, (A2) > (A1)^*∗*^ (4) (A1) > (B)^*∗*^, (A2) > (B)^*∗*^	None
Shi 2011 [24]	126 (42 : 42 : 42) →126 (42 : 42 : 42)	(A1) 67.90 ± 6.20 (NR)/27 : 15 (A2) 68.92 ± 6.11 (NR)/28 : 14 (B) 69.12 ± 5.66 (NR)/30 : 12	VD (NINDS-AIREN)	(A1) AA (A2) AA + Moxa	WM (Almitrine-Raubasine tablets 1T bid)	(1) MMSE (2) ADL (3) HDS (4) Clinical efficacy rate (TCM guideline on treatment of senile dementia)	(1) (A2) > (A1)^*∗*^, (A2) > (B)^*∗*^ (2) (A2) > (A1)^*∗*^, (A2) > (B)^*∗*^ (3) (A2) > (A1)^*∗*^, (A2) > (B)^*∗*^ (4) (A2) > (A1)^*∗*^, (A2) > (B)^*∗*^, (A1) > (B)^*∗*^	None

*AA + WM vs WM*								

Ai 2013 [25]	60 (30 : 30) →60 (30 : 30)	NR (41–68)/32 : 28	VD (DSM-IV)	AA + (B)	WM (Piracetam 0.8 g 1T tid)	Clinical efficacy rate (dementia symptoms)	(A) > (B)^*∗*^	NR
Liu 2016 [26]	93 (33 : 29 : 31) →90 (31 : 28 : 31)	(A) 71.23 ± 9.08 (60–90)/16 : 15 (B1) 71.57 ± 7.53 (60–88)/12 : 16 (B2) 71.13 ± 8.90 (61–91)/13 : 18	MCI MMSE > 17 (illiteracy), >20 (primary school group), >24 (middle school and above group) MoCA < 26	AA + (B1)	(B1) WM (Cinnarizine 25 mg tid, Methylcobalamin 0.5 mg qd, Piracetam 0.8 g tid, Salvia tablets 2T tid) (B2) Wait-list	(1) MMSE (2) MoCA	(1) at 12 months: (A) > (B2)^*∗*^, (B1) > (B2)^*∗*^/at 1, 3, 6 months: N.S (2) at 12 months: (A) > (B2)^*∗*^/at 1, 3, 6 months: N.S	None
Rodríguez-Mansilla et al. 2015 [27]	120 (40 : 40 : 40) →111 (40 : 35 : 36)	(A) 85.4 ± 5.9 (NR)/6 : 34 (B1) 85.8 ± 4.9 (NR)/9 : 26 (B2) 81.9 ± 5.9 (NR)/10 : 26	Elderly (>65 years) Dementia (DSM-IV) MMSE 0–20	AA + (B2)	(B1) relaxing massage + (B2) (B2) routine care (WM and physiotherapy)	(1) Structured questionnaire ((a) behavior alterations, (b) sleep disturbance, (c) participation in rehabilitation, (d) participation in eating) (2) Doloplus-2 scale (change) (3) CSDD (change) (4) Campbell scale (change)	(1) N.S, but at 2 months of follow-up: (d) (A) > (B1)^+^ (2) at 1 month: (A) > (B1)^*∗*^/at 2, 3 months, at 1 month of follow-up: (A) > (B1)^+^/at 2 months of follow-up: N.S (3) at 1, 2, 3 months, at 1 month of follow-up: (A) > (B1)^*∗*^/at 2 months of follow-up: N.S (4) at 1 month of follow-up: (A) > (B1)^*∗*^/at 1, 2, 3 months, at 2 months of follow-up: N.S	NR

*AA + HM vs HM*								

Chen et al. 2013 [28]	64 (32 : 32) →64 (32 : 32)	72.3 ± 11.6 (NR)/38 : 26	Elderly (>65 years) MCI (CCMD-2-R) MMSE 18–26 ADL 21–50	AA + HM	HM	(1) MMSE (2) ADL	(1) (A) > (B)^*∗*^ (2) (A) < (B)^*∗*^	None
X. Lu and S. Lu 2008 [29]	30 (15 : 15) →NR	NR/NR	60–75 years Dementia (ICD-10) MMSE 18–26 ADL 21–50	AA + HM	HM	(1) MMSE (2) ADL (3) Improvement rate (MMSE) (4) Improvement rate (ADL)	(1) (A) > (B)^†^ (2) (A) < (B)^†^ (3) (A) > (B)^†^ (4) (A) > (B)^†^	NR
Xiao et al. 2014 [30]	90 (30 : 30 : 30) →90 (30 : 30 : 30)	(A) 78.32 ± 5.47 (NR)/9 : 21 (B1) 75.68 ± 4.71 (NR)/10 : 20 (B2) 76.59 ± 5.08 (NR)/11 : 19	VCIND (criteria of expert consensus)	AA + HM	(B1) HM (B2) WM (Nimodipine 30 mg 1T tid)	(1) MMSE (2) MoCA (3) Serum 25-hydroxyvitamin D	(1) (A) > (B1)^†^, (A) > (B2)^*∗*^ (2) (A) > (B1)^*∗*^, (A) > (B2)^*∗*^ (3) (A) > (B2)^+^, (B1) > (B2)^+^	NR

AA: auricular acupressure; ADL: activities of daily living; CCMD: Chinese classification of mental disorders; CSDD: Cornell scale for depression in dementia; DSM: diagnostic and statistical manual of mental disorders; HDS: Hierarchic Dementia Scale; HIS: Hachinski Ischemic Scale; HM: herbal medicine; ICD: international classification of diseases; MCI: mild cognitive impairment; MMSE: Mini-Mental State Examination; MoCA: Montreal Cognitive Assessment; NINDS-AIREN: National Institute of Neurological Disorders and Stroke and Association-Internationale pour la Recherché et l'Enseignement Neurosciences; NR: not recorded; TCM: traditional Chinese medicine; VCIND: vascular cognitive impairment, no dementia; VD: vascular dementia; WM: Western medication; ^*∗*^*P* < 0.05; ^+^*P* < 0.01; ^†^statistical tests were not performed; N.S: not significant between interventions.

**Table 2:** Details of the auricular acupuncture method.

Study ID	Acupoints	Location of points used	Material	Material replacement period	Frequency and duration of self-acupressure	Treatment period	Follow-up assessment
*AA vs WM*							

Chen et al. 2009 [22]	*Shen men*, brain, kidney, occiput	bilateral	*Vaccaria* seeds	Once a day	5 times/day, 5 minutes/session	12 weeks	NR
Kuang et al. 2012 [23]	Forehead, subcortex, temple	unilateral	*Vaccaria* seeds	Once every 2 days	3 times/day	12 weeks	NR
Shi 2011 [24]	Brain, kidney, heart, spleen	bilateral	*Vaccaria* seeds	Once every 3 days	3 times/day	12 weeks	12 weeks

*AA + WM vs WM*							

Ai 2013 [25]	*Shen men*, brain, kidney, heart	bilateral	*Vaccaria* seeds	Once a day	5 times/day, 5 minutes/session	3 months	NR
Liu 2016 [26]	*Shen men*, brain, kidney, heart, forehead, subcortex	bilateral	*Vaccaria* seeds	2-3 times/week	NR	12 months	NR
Rodríguez-Mansilla et al. 2015 [27]	*Shen men, *heart	NR	*Vaccaria* seeds	Every 15 days	NR	3 months	2 months

*AA + HM vs HM*							

Chen et al. 2013 [28]	*Shen men*, kidney, heart, forehead, subcortex	NR	*Vaccaria* seeds	NR	2 times/day, 1-2 minutes/session	3 months	NR
X. Lu and S. Lu 2008 [29]	*Shen men*, kidney, heart, forehead, subcortex, etc.	NR	NR	NR	NR	45 days	NR
Xiao et al. 2014 [30]	*Shen men*, brain, kidney, heart, spleen	NR	NR	NR	3 times/day, 3 minutes/session	6 months	NR

AA: auricular acupressure; HM: herbal medicine; NR: not recorded; WM: Western medication.

## Supplementary Materials

Supplementary Materials Supplementary Materials include the search terms for each database described in Materials and Methods and PRISMA checklist of this review. Supplement 1: search strategy used in all databases; Supplement 2: PRISMA checklist.
