# Supplementary material for: Efficacy and Safety of Auricular Acupuncture for Cognitive Impairment and Dementia: A Systematic Review
Source: Evid Based Complement Alternat Med. 2018 May 31;2018:3426078. doi: 10.1155/2018/3426078 (PMC6000857; doi:10.1155/2018/3426078)
Supplement: Supplementary Materials — include the search terms for each database described in Materials and Methods and PRISMA checklist of this review. Supplement 1: search strategy used in all databases; Supplement 2: PRISMA checklist. [file 3426078.f1.zip › Supple 1.docx]

**Supplementary Material**

**Supplement 1. Search Strategy Used in All Databases**

*PubMed, Cochrane Central Register of Controlled Trials (CENTRAL), AMED, CINAHL, KoreaMed*

(Acupuncture, Ear OR auricular therapy OR auriculotherapy OR ear acupuncture OR auricular acupuncture OR ear acupressure OR auricular acupressure OR auricular point OR ear point) AND (hippocampus OR memory OR cognition OR cognitive OR dementia OR amnesia OR amnestic OR MCI)

*EMBASE*

(’Acupuncture, Ear’ OR ’auricular therapy’ OR ’auriculotherapy’ OR ’ear acupuncture’ OR ’auricular acupuncture’/exp OR ’auricular acupuncture’ OR ’ear acupressure’ OR ‘auricular acupressure’/exp OR 'auricular acupressure' OR ’auricular point’ OR ’ear point’) AND ('hippocampus'/exp OR 'hippocampus' OR 'memory'/exp OR 'memory' OR 'cognition'/exp OR 'cognition' OR 'cognitive' OR 'dementia'/exp OR 'dementia' OR 'amnesia'/exp OR 'amnesia' OR 'amnestic' OR 'MCI' OR 'mild cognitive impairment'/exp OR 'mild cognitive impairment' OR 'mild cognitive defect'/exp OR 'mild cognitive defect')

*psycARTICLES*

(“Acupuncture, Ear” OR “auricular therapy” OR “auriculotherapy” OR “ear acupuncture” OR “auricular acupuncture” OR “ear acupressure” OR “auricular acupressure” OR “auricular point” OR “ear point”) AND (“hippocampus” OR “memory” OR “cognition” OR “cognitive” OR “dementia” OR “amnesia” OR “amnestic” OR “MCI”)

*CNKI*

(SU='耳针' + '耳鍼' + '耳压' + '耳穴' + 'auricular therapy' + 'auriculotherapy' + 'ear acupuncture' + 'auricular acupuncture' + 'ear acupressure' + 'auricular acupressure' + 'auricular point' + 'ear point')

AND (SU='海马' + '记忆' + '认知' + '痴呆' + '健忘' + '善忘' + 'hippocampus' + 'memory' + 'cognition' + 'cognitive' + 'dementia' + 'amnesia' + 'amnestic' + 'MCI')

*WanFang data*

(“耳针” + “耳鍼” + “耳压” + “耳穴”) * (“海马” + “记忆” + “认知” + “痴呆” + “健忘” + “善忘”)

*OASIS*

(이침 OR 이혈 OR 이압 OR Acupuncture, Ear OR auricular therapy OR auriculotherapy OR ear acupuncture OR auricular acupuncture OR ear acupressure OR auricular acupressure OR auricular point OR ear point OR 耳穴 OR 耳鍼) AND (해마 OR 인지 OR 기억 OR 치매 OR 건망 OR hippocampus OR memory OR cognition OR cognitive OR dementia OR amnesia OR amnestic OR MCI OR 海馬 OR 記憶 OR 認知 OR 癡呆 OR 健忘 OR 善忘)

*KTKP*

(이침 | 이혈 | 이압 | Acupuncture, Ear | auricular therapy | auriculotherapy | ear acupuncture | auricular acupuncture | ear acupressure | auricular acupressure | auricular point | ear point | 耳穴 | 耳鍼) & (해마 | 인지 | 기억 | 치매 | 건망 | hippocampus | memory | cognition | cognitive | dementia | amnesia | amnestic | MCI | 海馬 | 記憶 | 認知 | 癡呆 | 健忘 | 善忘)

*KISS*

(이침 OR 이혈 OR 이압) AND (인지 OR 기억 OR 치매 OR 건망)
